# Assessment of antimicrobial and wound healing effects of Brevinin-2Ta against the bacterium *Klebsiella pneumoniae* in dermally-wounded rats

**DOI:** 10.18632/oncotarget.22797

**Published:** 2017-11-30

**Authors:** Siqin Liu, Qilin Long, Yang Xu, Jun Wang, Zhongwei Xu, Lei Wang, Mei Zhou, Yuxin Wu, Tianbao Chen, Chris Shaw

**Affiliations:** ^1^ Natural Drug Discovery Group, School of Pharmacy, Queen’s University Belfast, United Kingdom; ^2^ Department of General Surgery, The First Affiliated Hospital of Tianjin University of Traditional Chinese Medicine, Tianjin, China; ^3^ Center Laboratory of Logistics University of Chinese People’s Armed Police Forces, Tianjin, China

**Keywords:** AMP, brevinine, inflammation, angiogenesis, Klebsiella pneumoniae

## Abstract

Antimicrobial peptides (AMPs) are regarded as promising alternatives for antibiotics due to their inherent capacity to prevent microbial drug resistance. Amphibians are rich source of bioactive molecules, which provide numerous AMPs with various structures as drug candidates. Here, we isolated and identified a novel AMP Brevinin-2Ta (B-2Ta) from the skin secretion of the European frog, *Pelophylax* kl. *esculentus*. *In vitro* studies revealed that it showed broad antimicrobial activities against *S. aureus*, *E. coli* and *C. albicans* with low cytotoxicity to erythrocytes. Furthermore, we examined the anti-inflammation effect *in vivo* by using *Klebsiella pneumoniae*-infected Sprague-Dawley (SD) rats. The wound closure outcomes revealed that B-2Ta effectively restrained the bacterial infection at a dose of 10 times minimal inhibitory concentration (MIC) during the 14 days of the wound healing process. Ultra-structure analyses showed that B-2Ta caused structural damage to the microorganism, and bacterial culture found that the number of microbes was significantly reduced by the end of treatment. Immunohistochemistry for the inflammatory marker IL-10 and the endothelial cell marker CD31 suggested positive effects on inflammatory status and epithelial migration and angiogenesis following treatment of the infected granulation tissues with B-2Ta. These results exhibited the continuous phase of inflammation reduction and wound healing acceleration in the B-2Ta-modulated re-epithelialisation of *K. pneumoniae* infected rats. Taken together, these data demonstrated that B-2Ta has great potential to be developed as antibacterial agents in clinic.

## INTRODUCTION

Bacterial infections occur with injuries, acute or chronic ulcers or after surgical treatment. Pathogenic organisms can invade the whole body, attach to cells and multiply in the host, and in some cases, the immune system fails to prevent the infections caused by these pathogens. Bacteria exist on the surface of the skin and infections are commonly initiated following injuries or immunity depression. Skin wounds are immediate causes which lead the microorganisms to break through the skin into the circulatory system and usually occur with trauma and ulcers, for example, diabetic foot gangrene, which is commonly infected by the Gram-positive bacteria *Staphylococcus aureus*, *β*-haemolytic streptococci and the Gram-negative *Klebsiella pneumonia* (*K. p*).

*K. p* is one of the most common pathogens in nosocomial infections, and it has become increasingly multidrug-resistant. It causes serious pulmonary and urinary tract infections in hospitalised patients, patients with diabetes, alcoholism, and chronic lung disease are also susceptible for *Klebsiella* [1]. Large numbers of clinical cases with chronic ulcers are reported to be infected by multiple microbes including *K. p*. Point mutations and resistance genes acquisition through lateral gene transfer are two major causes that lead to the development of multi-drug resistant *K. p* [2]. Multi-faceted factors are involved in *K. p* infections in a coordinated manner [3].

When bacteria invade the internal environment through dermal injuries, proliferation and reproduction of bacterial factors occur first, then the inflammatory response is induced and followed by the immune response. The wound healing process is divided into 4 phases: homeostasis, inflammation, granulation (proliferation) and remodelling. During periods of rebuilding extracellular matrix, fibroplasia and angiogenesis, significant molecular networks, such as adhesion molecules, proteinases, cytokines, chemokines and growth factors, are involved [4]. Endogenous microbes can infect the surgical wounds from the organs inside the body or exogenous microbes on the skin/in the air, in particular for diabetic patients who are taking corticosteroids or who just had surgery. The general treatment for wound infections is antibiotics and surgery. In such cases, selective and effective antibiotic alternatives, especially those with the potential to overcome drug resistance, are a valuable therapeutic option.

In this study, a novel AMP named B-2Ta was isolated and identified from the European frog *Pelophylax* kl. *esculentus*, it showed broad spectrum antimicrobial properties and anti-proliferative effects against several bacterial strains with low toxicity. In a pre-clinical *in vivo* study, B-2Ta effectively inhibited the inflammatory responses and promoted angiogenesis in SD rats. Therefore, we report that B-2Ta is a potential therapeutic agent for effectively promoting *K. p*-infected wound healing.

## RESULTS

### Molecular cloning of B-2Ta precursor cDNA from *pelophylax* kl. *esculentus* skin secretion

**Figure 1:**
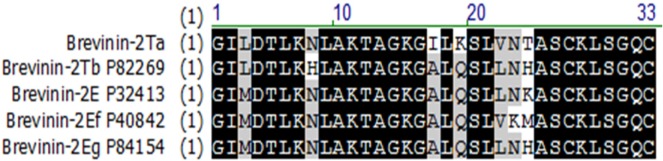
Alignment of B-2Ta with its closest homologues identified via BLAST analyses of the NCBI database The conserved regions are highlighted in black; grey areas indicate the less conserved residues, sites of amino acid which have remarkable differences are shown in the blank. Database accession numbers are listed following each named homologue.

The B-2Ta precursor encoding cDNA was repeatedly cloned and replicated by RACE PCR procedures from the skin secretion derived cDNA library. The open reading frame of this cDNA consisted of 74 amino acid residues, of which the first 22 residues were a putative signal peptide domain, a two-amino acid (-Lys40-Arg41-) propeptide convertase processing motif that is responsible for enzyme digestion [5], and a single copy of a 33-amino acid residue mature peptide (GILDTLKNLAKTAGKGILKSLVNTASCKLSGQC) was located at the C-terminal (Supplementary Figure 1). BLAST results indicated that a single residue substitution existed between the homologous precursor sequences from *Rana. temporaria* and *Pelophylax* kl. *esculentus*. Also, the novel B-2Ta isolated from *Pelophylax* kl. *esculentus* shared 85% identity with five others mature brevinin-2 peptides isolated from three congeneric species which were *Pelophylax ridibundus* (brevinin-2Eg, accession no. P86154; brevinin-2Ef, accession no. P86153), *Pelophylax* kl. *esculentus* (brevinin-2E, accession no. P32413), and *Rana temporaria* (brevinin-2Tb, accession no. P82269) (Figure 1). Analyses of cDNAs coding for the corresponding precursors indicated that a single copy of the mature peptide sequence was preceded by a dibasic cleavage site and followed by a stop codon [6]. The nucleotide sequence of the cDNA encoding the novel antimicrobial Brevinin-2Ta peptide precursor from the skin secretion of Pelophylax kl. esculentus, has been deposited in the EMBL Nucleotide Sequence Database under the accession code: LT615139.

### Isolation and structural characterisation of B-2Ta

**Figure 2:**
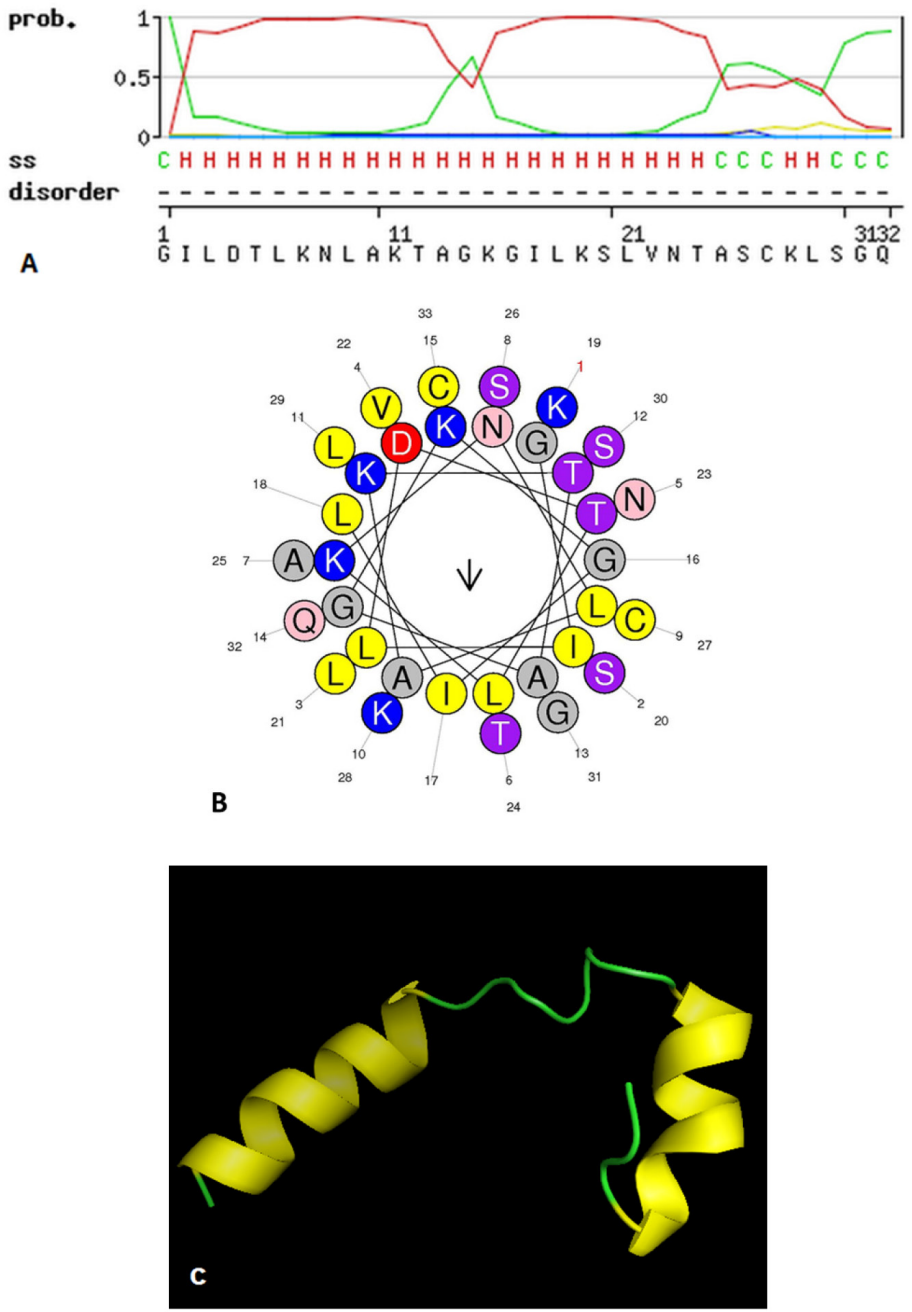
The spatial characteristics of the peptide simulated in three aspects, **(A)** SWISS-MODEL assessment of secondary structure of the mature peptide B-2Ta. C=random coils and H=helix. **(B)** Helical wheel plot of B-2Ta, indicates its amphipathic properties, the residues L9, I2, L6, I17, L3, L21 occupied one side of the helix and the cationic amino groups K7, K11, K15, K19 presented on the other side. **(C)** The 3D model graph of B-2Ta predicted via the SWISS-MODEL ExPASY server and visualised by the PyMOL platform. Random chains connected two modules of the helical column.

The putative mature peptide B-2Ta was identified in a fraction generated by reverse phase HPLC (rp-HPLC) of *Pelophylax* kl. *esculentus* skin secretion (Supplementary Figure 2). MALDI-TOF mass spectrometry, using a Perseptive Biosystems Voyager DE system in linear mode, identified this single peptide with an m/z of 3347.01(data not shown). Electrospray ion-trap MS/MS established the primary structure of the peptide B-2Ta as: GILDTLKNLAKTAGKGILKSLVNTASCKLSGQC (Supplementary Figure 3). Previous studies reported that peptides belonging to this family possess an intramolecular disulphide bridge at the C-terminus [6]. The unequivocally establishment of the primary structure of mature B-2Ta was achieved using LCQ fleet™ electrospray ion-trap mass spectrometry and molecular cloning strategy. Solid Phase Peptide Synthesis was employed to obtain peptide replicates for functional testing. The MALDI-TOF was used for the purity assessment of the synthetic peptide, which constituted >90% of the total components. Secondary structure, predicted using the SWISS-MODEL database (Figure 2A), indicated that the α-helix occupied the main part of B-2Ta, while two cysteines of the nonapeptide tail at the C-terminal, created a cyclic structure by a disulphide bridge. The helical wheel plots are shown in Figure 2B. The aggregated distribution of cationic and hydrophobic residues on the opposite surfaces of the helix forms an amphipathic structure which confirmed the potential of the peptide to interact with cellular membrane structures. The 3D mimic pattern (Figure 2C) visualised the structural mode of B-2Ta which consisted of two separate parts of a helical unit connected to a random amino acid chain.

### Evaluation of antimicrobial and haemolytic effects of B-2Ta

**Figure 3:**
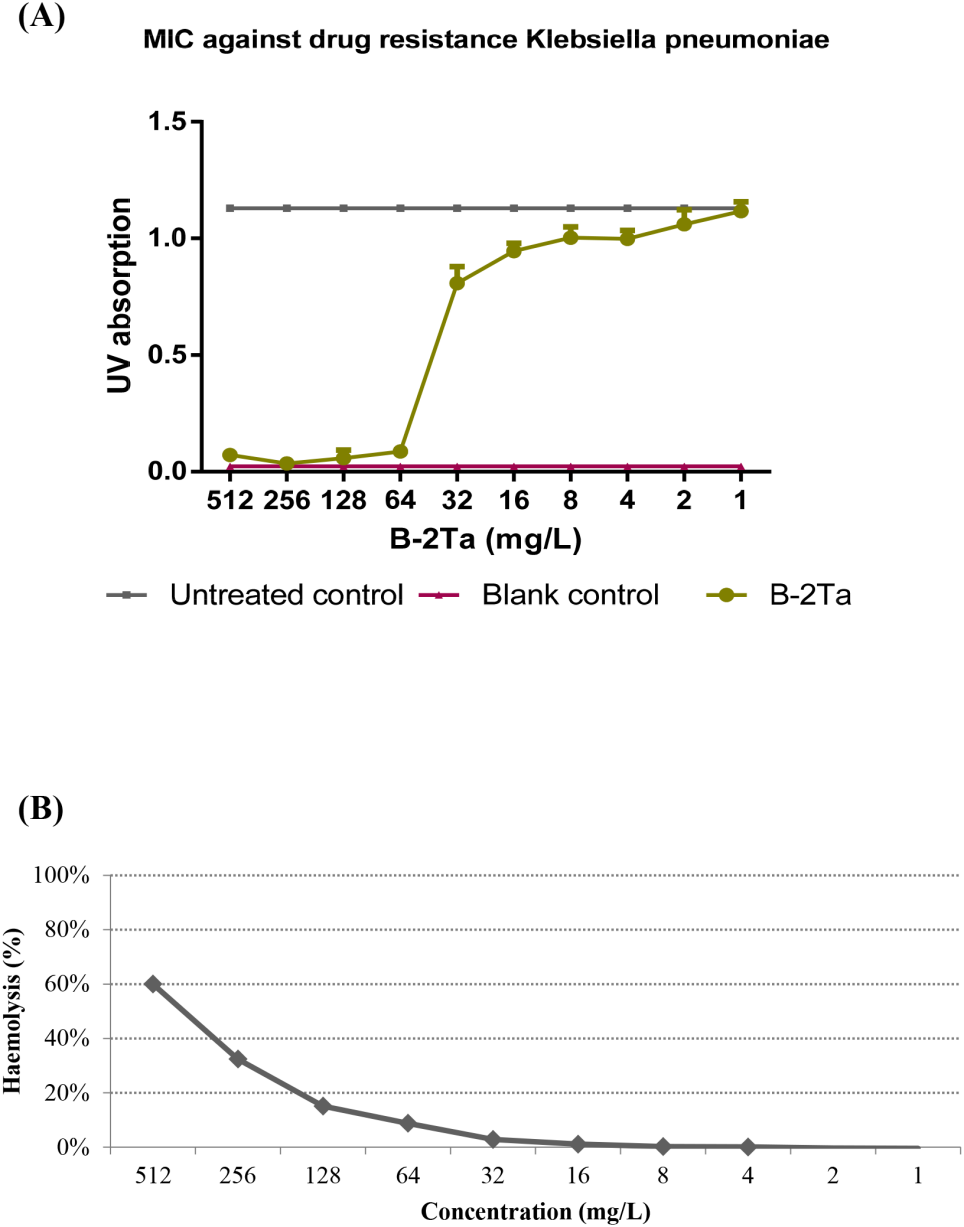
**(A) Antimicrobial activity of B-2Ta against** *K. p*. The MIC value was 64 mg/L, IC_50_=38.1 mg/L. **(B)** Haemolytic activity of B-2Ta. The maximal concentration caused 60% red cells lysis, while this activity significantly decreased to less than 10% at MICs of B-2Ta towards each organism.

The data obtained from minimal inhibitory concentration (MIC) and minimal bactericidal concentration (MBC) assays on the synthetic peptide B-2Ta were compared to those of melittin and ampicillin [7] (Table 1). MICs of B-2Ta were 64 mg/L (20 μM) against *S. aureus*, 32 mg/L (10 μM) against *E. coli*, and 64 mg/L (20 μM) against *C. albicans*. As a well-defined broad-spectrum antibacterial honey bee venom peptide, melittin possessed distinct cytotoxicity (>70% haemolysis) at the MICs. Ampicillin exhibited strong effects against *S. aureus* (0.0625 mg/L) and *E. coli* (0.125 mg/L), whereas it was ineffective towards *C. albicans*. The MBCs of the natural peptides, B-2Ta and melittin, were found to be two-fold higher compared with their MICs. However, MBCs of ampicillin against *S. aureus* and *E. coli* were 256 and 64-fold increase over the MICs, respectively. The haemolytic effect of B-2Ta are shown in Figure 3B, it displayed that 60% of horse blood erythrocytes were lysed at the maximal concentration of peptide (512 mg/L), while the peptide possessed only low level of haemolysis (<10%) at the MICs against *S. aureus*, *E. coli* and *C. albicans* (Table 1).

**Table 1:** Mean MICs and MBCs of the B-2Ta, melittin and ampicillin against the *S. aureus*, *E. coli* and *C. albicans*

	*S. aureus*	*E. coli*	*C. albicans*
	**MICs (mg/L & μM) and haemolysis (%) at corresponding MIC values**		
B-2Ta	64 mg/L (20 μM) (9%)	32 mg/L (10 μM) (3%)	64 mg/L (20 μM) (9%)
Melittin	8 mg/L (2.8 μM) (73.8%)	16 mg/L (5.6 μM) (75.2%)	8 mg/l (2.8 μM) (73.8%)
Ampicillin	0.0625 mg/L (0.18μM) (0%)	0.125 mg/L(0.36μM) (0%)	NE
		**MBCs (mg/L &μM)**	
B-2Ta	128 mg/L (40 μM) (15%)	64 mg/L (20 μM) (9%)	128 mg/L (40 μM) (15%)
Melittin	16 mg/L (5.6μM)	32 mg/L (11.2μM)	16 mg/L (5.6μM)
Ampicillin	16 mg/L (45.8μM)	8 mg/L (22.9μM)	NE

Meanwhile, the antimicrobial assessment of B-2Ta against *K. pneumoniae in vitro* showed that the MIC value was 64 mg/L, and IC_50_ value was 38.1 mg/L (Figure 3A).

### B-2Ta improves *K. pneumoniae*-infected wound healing in SD rats

**Figure 4:**
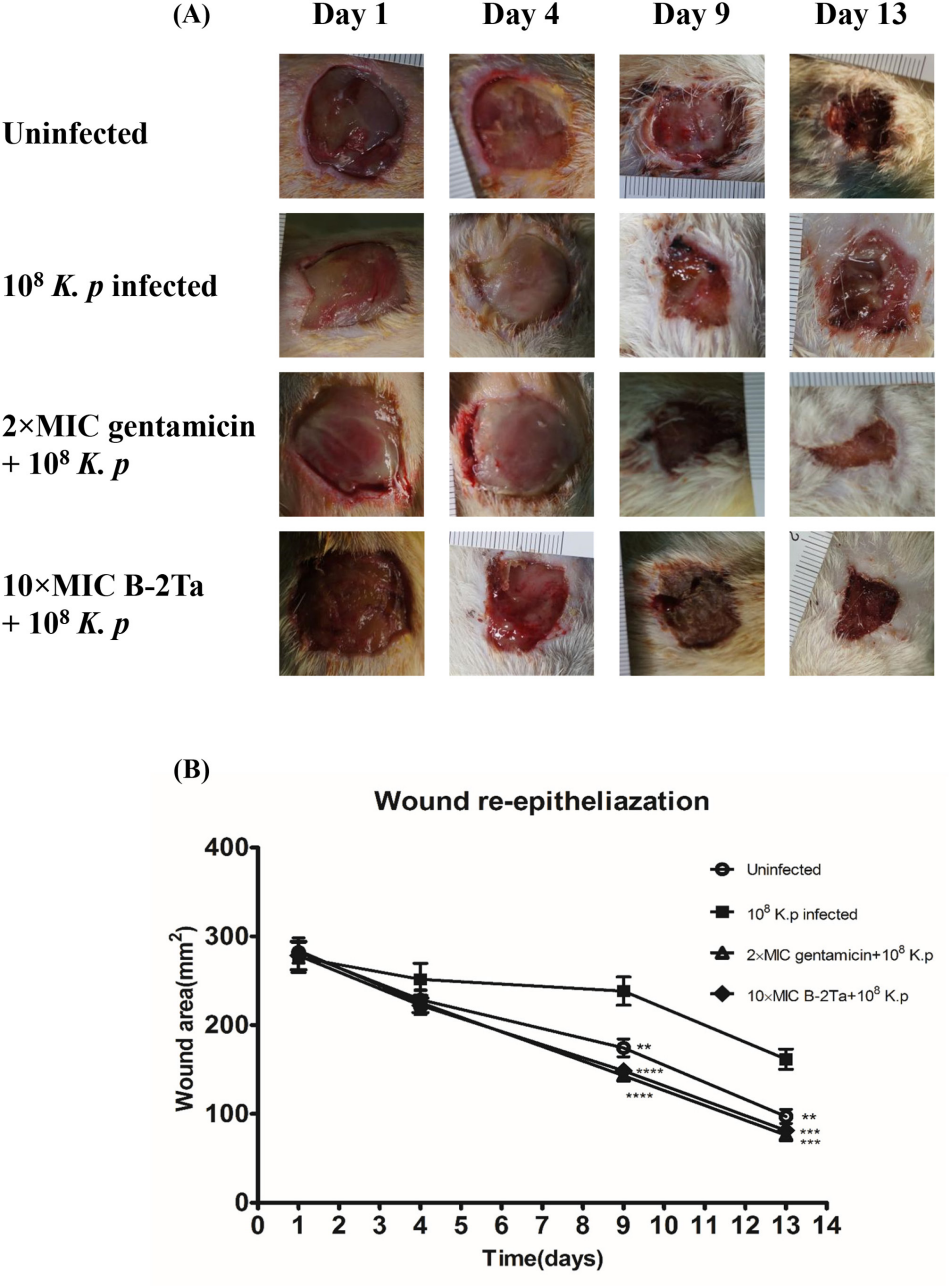
B-2Ta improved *K. p*-infected wound healing **(A)** The morphological changes in wound area of uninfected, 10^8^ *K. p*-infected, 2×MIC gentamicin-treated and 10×MIC B-2Ta-treated groups at 1, 4, 9, 13 days. **(B)** The re-built wound areas of four experimental groups, which were calculated by normalised with initial wounding area on day 0. Data were compared using ANOVA (Bonferroni test) with the SPSS statistical software 18.0. Significance: ^****^P<0.0001 vs *K. p*-infected group, ^***^P<0.001 vs *K. p*-infected group, ^**^P<0.005 vs *K. p*-infected group.

Wound closure assessment was based on the areas of the last generated tissues covering the dermal wounds. Figure 4A shows the fresh impaired wound of a SD rat and the healing degree on days 1, 4, 9 and 13. Rats in the uninfected control group appeared to be slightly purulent on the wound surface at day 4, which was normal during the inflammation phase. Other infected groups exhibited a slight bleeding thick mixture of layers of organisms and purulence. Statistical analyses showed that *K. p*–infected rats regenerated 5% less tissues than the other groups in the first four days (Figure 4B). During the proliferation and maturation phases, the granular tissues that contained collagen and extracellular matrix, were rebuilt and angiogenesis developed. At day 9, the *K. p*–infected group (group B) appeared to develop severe inflammation, and wound healing was barely observed. 10×MIC B-2Ta-treated group (group D) showed a mass of maturation and dark coloured granulation, suggesting a rebuilt and angiogenesis process. The 2×MIC gentamicin-treated group (group C) presented pinker granular tissues and less inflammatory purulence. On the 13th day, all the wounds began to scab with thinner moist areas and less inflammation except the *K. p*–infected group (group B), where the 10×MIC B-2Ta-treated group (group D) displayed comparable wound heal rate (55.4%) with 2×MIC gentamicin-treated group (group C) (56.7%), while the uninfected group (group A) exhibited a slightly lower heal rate (48.8%). The morphological measurement showed the notable improvement of 10×MIC B-2Ta-treated rats during the period of re-epithelialisation compared with the non-interventional control (group B), which was at the same level compared with conventional antibiotics. Supplementary Table 2 summarises the marks of scores for the general morphological features of each group during the experimental period.

### B-2Ta decreases *K. pneumoniae* counts in wound area

**Figure 5:**
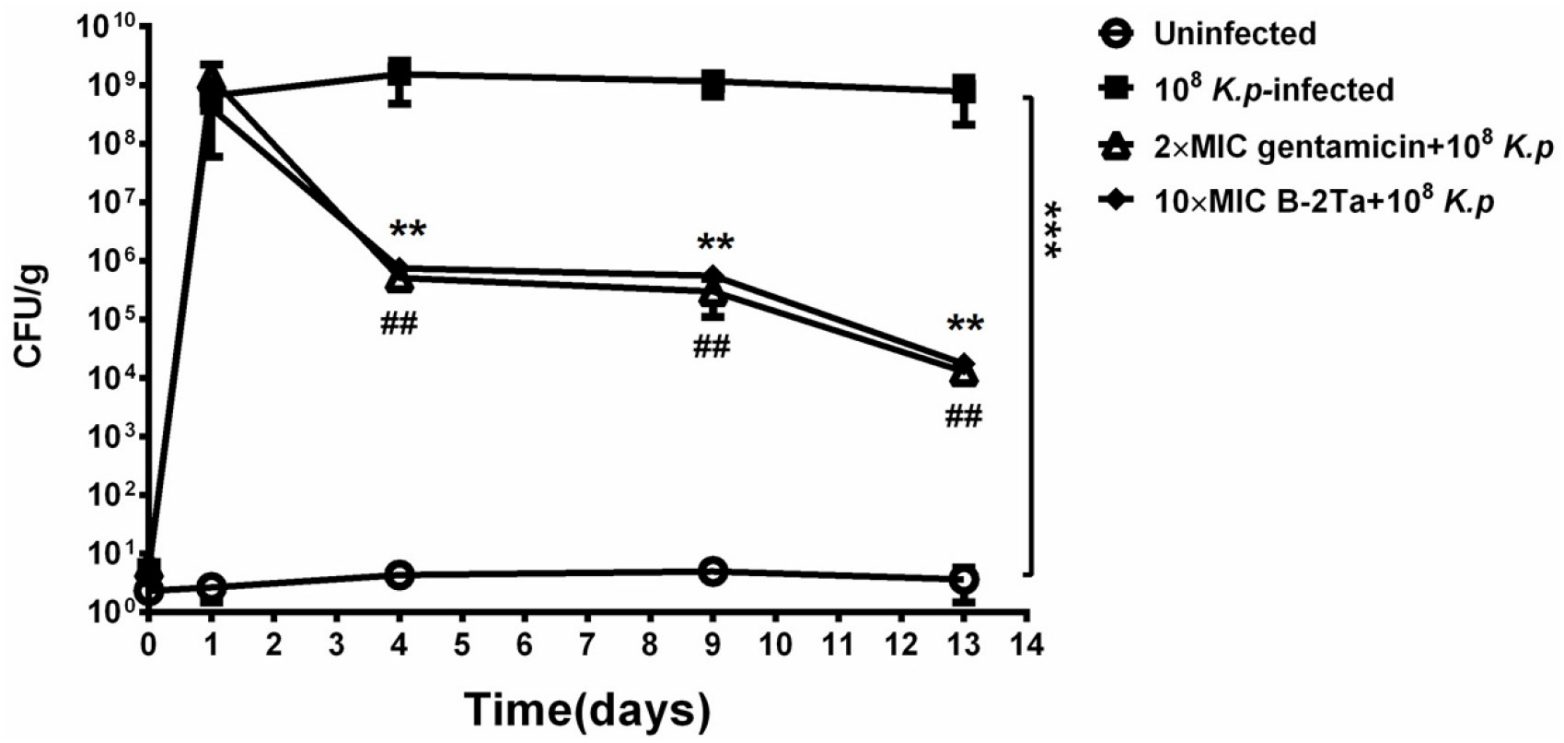
Quantitative bacterial culture of excised tissues on day 1, 4, 9, 13 after infection Significance: ^##^ or ^**^P<0.01 vs *K. p*-infected group.

B-2Ta and gentamicin exhibited MICs of 64 mg/L and 32 mg/L against *K. p* respectively. To assess the wound healing efficiency, the quantity of bacteria was determined as colony forming units / gram tissue (CFU/g). Figure 5 showed that the number of bacteria in all infected groups (group B, C, D) were all at the same level (7.95×10^8^± 2.30×10^8^ CFU/g) after inoculation (day 1). After the treatment of 2×MIC gentamicin and 10×MIC B-2Ta for 13 days, a significant reduction was observed in 2×MIC gentamicin-treated group with 1.85×10^4^±0.35×10^4^ CFU/g and in 10×MIC B-2Ta-treated group with 1.88×10^4^±0.73×10^4^ CFU/g. The bacterial culture screening of the exudate samples from wound areas at different time points reflected the same declining trend of the numbers of colonies (Supplementary Figure 3). Specifically, the data showed that the numbers of bacterial colonies were maintained at a high level on day 4 except for the uninfected group, while the microbial proliferation of 2×MIC gentamicin and 10×MIC B-2Ta treated groups became sparser on day 9, and prominent decline appeared on day 13.

**Figure 6:**
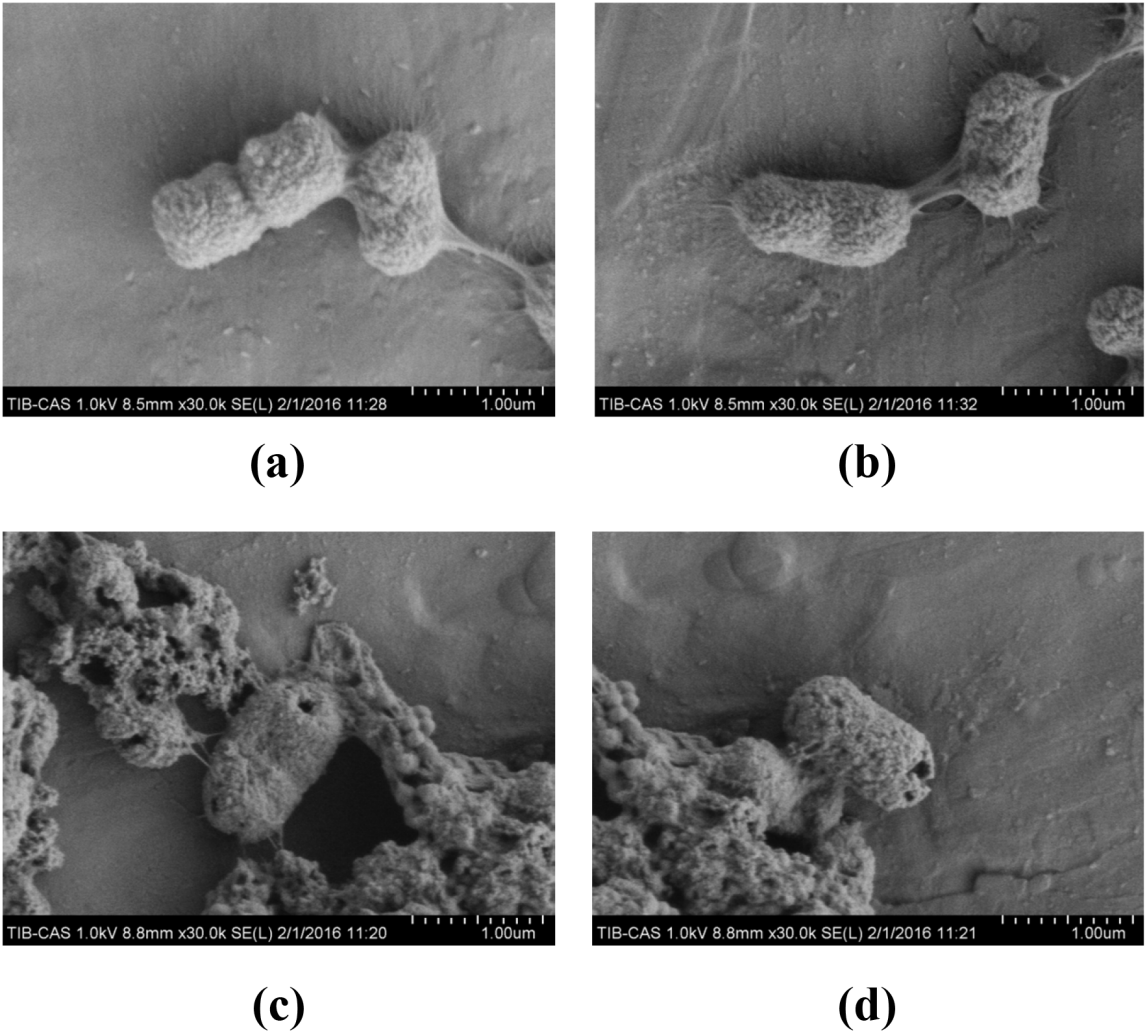
SEM micrographs of infected control **(a, b)**, 2×MIC gentamicin-treated **(c)** and 10×MIC B-2Ta-treated samples of clinical *K. p*. (a, b) The bacteria presented as rod-shaped, with integrated fimbriae around the enclosures. **(c, d)** The treatment of 2×MIC gentamicin or 10×MIC B-2Ta led to rapid transmembrane pore formation close to the polar and septal regions and breakdown of the integrity of bacterial surfaces.

Meanwhile, the morphology changes of bacteria were observed by using scanning electron microscopy (SEM). The graphs in Figure 6 showed that the normal bacteria were rod-shaped, with integrated fimbriae around the enclosures. When treated with B-2Ta (10×MIC=0.64 mg/ml) or gentamicin (2×MIC=64 mg/L), bacterial membranes were disrupted, causing the pore formation and the breakdown of structural integrity.

### B-2Ta improves the skin re-epithelisation, granulation and collagen deposition in wound area

**Figure 7:**
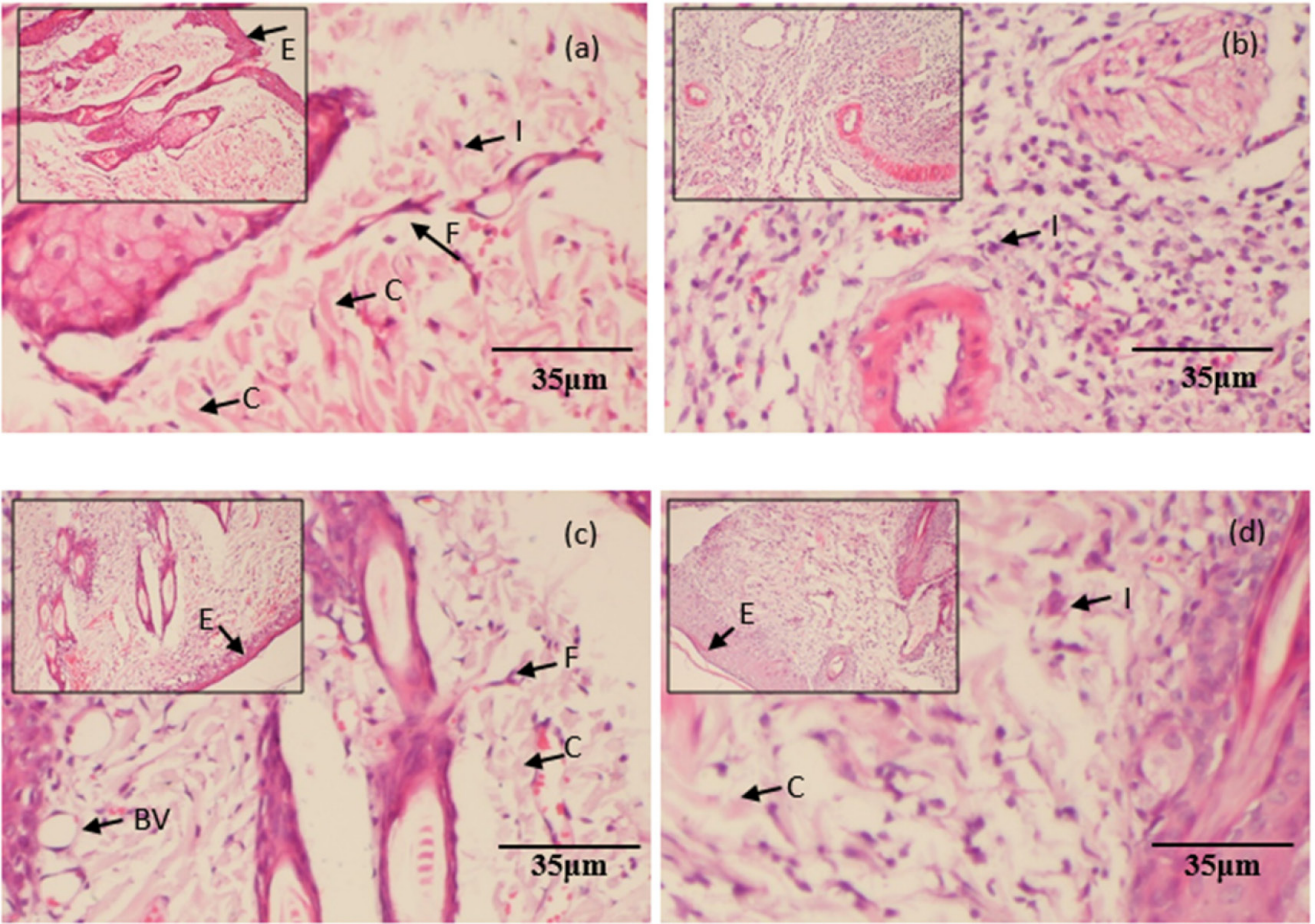
H&E stained histopathological sections of granulation/healing tissues The tissues were from **(a)** uninfected control, **(b)** 10^8^ *K. p*-infected group, **(c)** 2×MIC gentamicin-treated group, and **(d)** 10×MIC B-2Ta-treated group, collected on day 9 after infection. I: inflammation, F: fibroblasts, C: collagen, E: epithelial layer, BV: blood vessels.

Figure 7 displayed the haematoxylin and eosin (H&E) stained sections of granulation/healing tissue from different groups with or without treatment of gentamicin or B-2Ta. The uninfected control (group A) showed less inflammatory cell migration and integrated epithelial layer. The *K. p*-infected control (group B) showed a typical infection process with thin granulated tissue, sparse fibroblasts, and thick inflammatory cells. Meanwhile, the vessels are shapeless. Compared with the control groups, the 2×MIC gentamicin-treated group (group C) has improved healing process with regular epithelial layers and abundant integrated vessel circles presented, also, the fibroblast and collagens are clear in the wound sections. The inflammation status reflected that the 10×MIC B-2Ta-treated wounds (group D) contained more smooth collagens and fibroblasts compared with the infected control, and the regular epithelial layers and integrated vessel circles has been improved.

### B-2Ta enhances epithelial migration and angiogenesis

**Figure 8:**
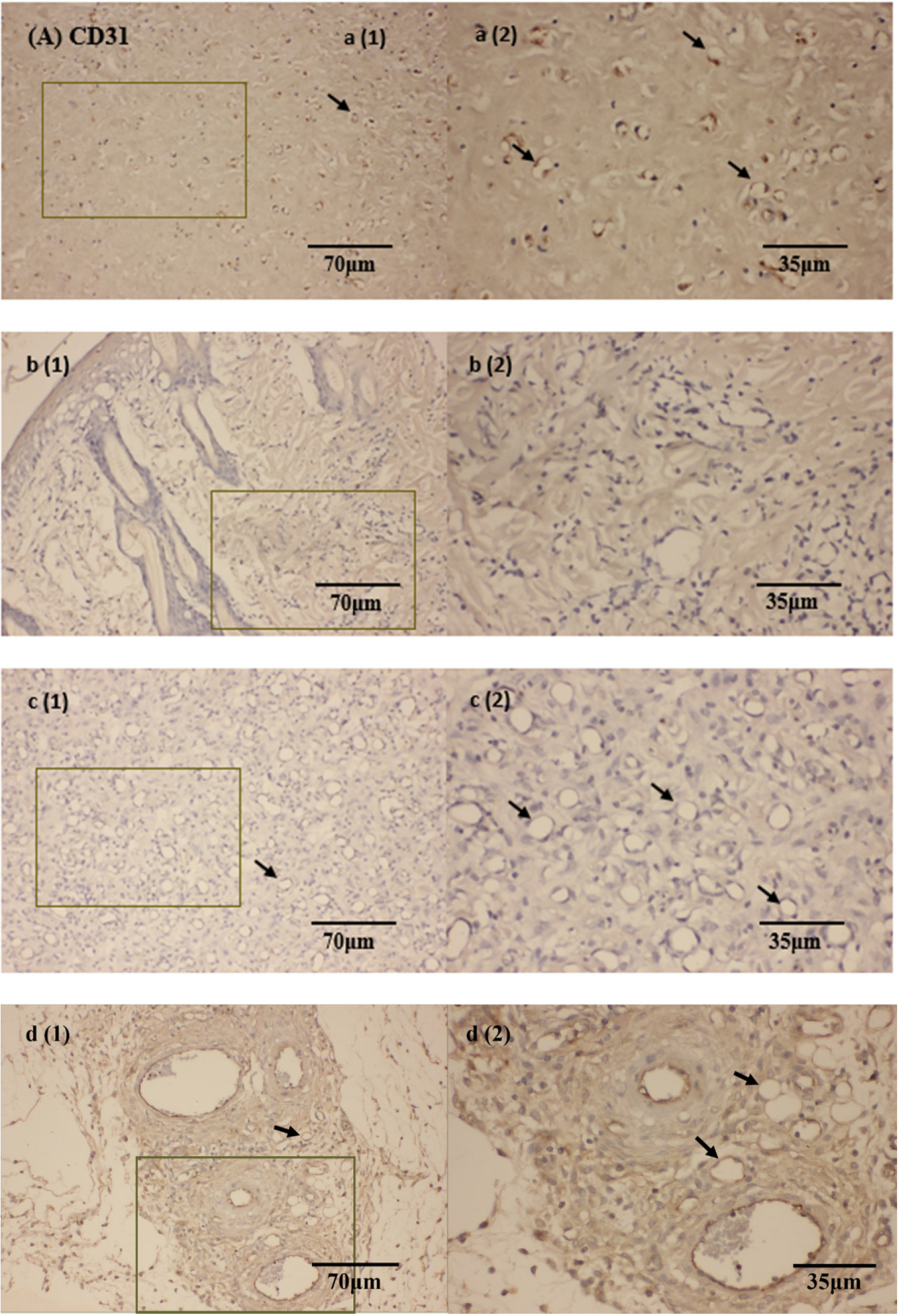

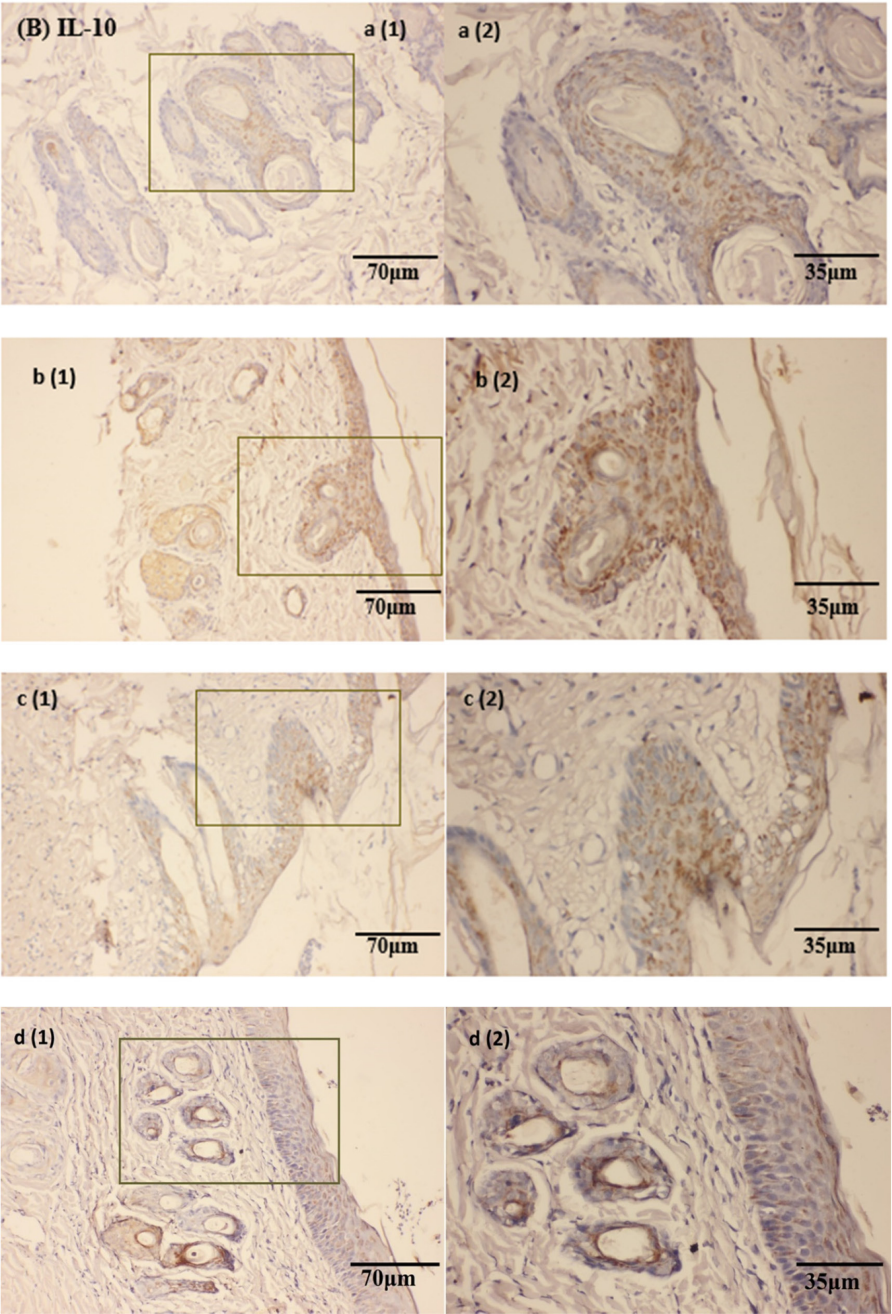

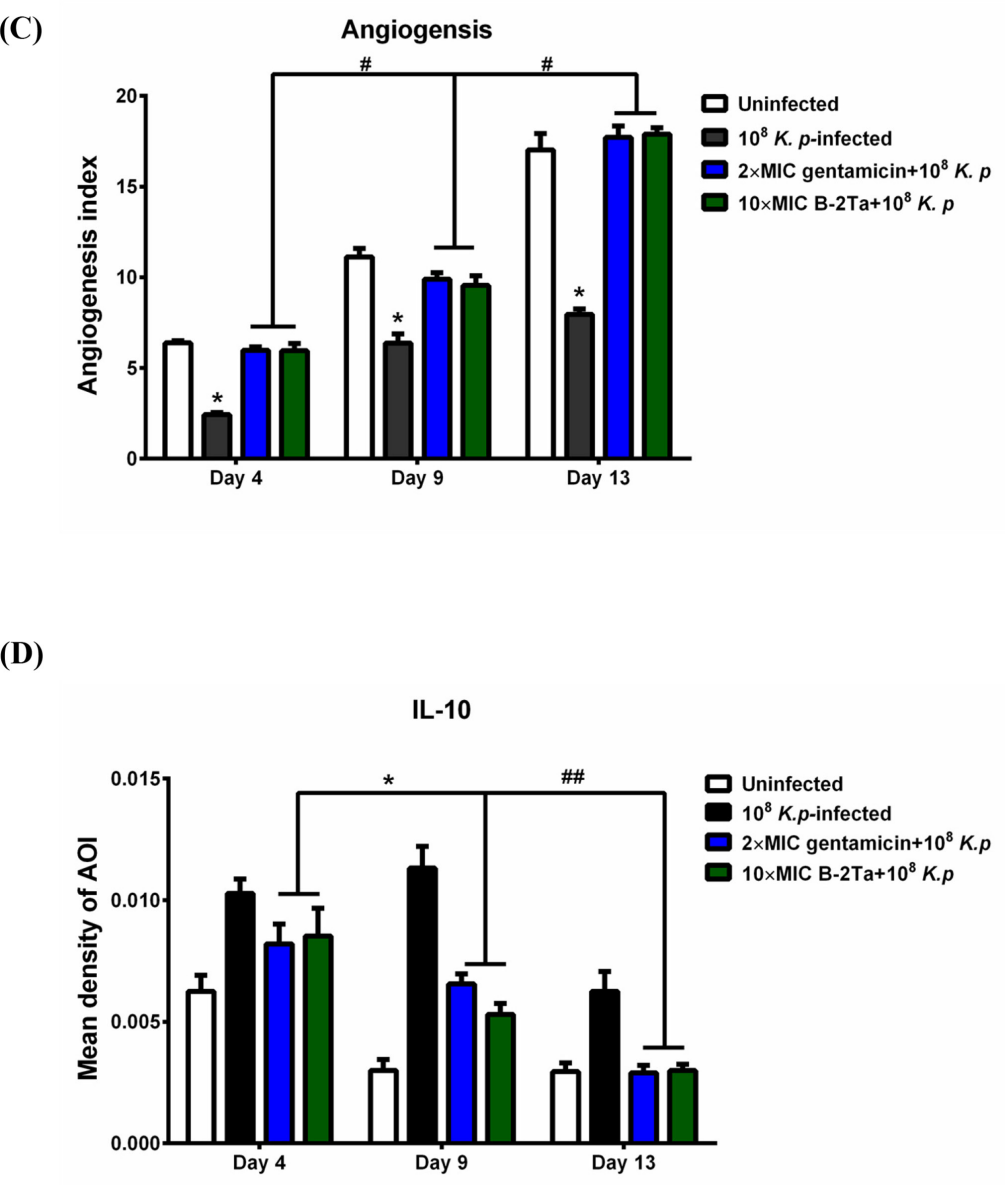
**(A)** CD31 and **(B)** IL-10 immunohistochemical (IHC) staining of skin wounds on day 9 after inoculation of *K. p*. Sections showed the intensity of protein expression of (a) uninfected control, (b) 10^8^ *K. p*-infected group, (c) 2×MIC gentamicin-treated group and (d) 10×MIC B-2Ta-treated group. **(C)** CD31, angiogenesis index=average vessel counts per field. **(D)** The intensity of positive stains was assessed by a mean density of the AOI (area of interest). Data are expressed as mean ± SEM of three sections. The arrows represent the endothelial CD31 positive blood vessels in graphs of (A). Significance: ^*^ P<0.05 vs uninfected group or between indicated groups, ^#^ p<0.05 between indicated groups.

Photomicrographs in Figure 8 A showed the CD31 expression in tissues with wound edge and the distribution of blood capillaries on day 9. The arrows indicated that the capillaries were present as cyclic sections, according to which, we can regard the regular and integrated circular rings as positive microvessels, which are normally constituted by 3-4 cells (with a visualised nucleus) and associated with CD31 accumulation, the positive vessels represented an epithelial regeneration status. In Figure 8A, the *K. p*-infected control group (group B) appeared to have incomplete edges with few and irregular capillary sections, which was relatively poor re-epithelial conditions; the uninfected control group (group A) contained well-shaped vessels and higher density of CD31 staining, indicating active angiogenesis in its proliferation phases; the 2×MIC gentamicin-treated group and 10×MIC B-2Ta-treated group (group C, D) started to heal in a middle degree compared with the control groups, which presented well-shaped vessels and slightly lower expressed CD31 levels than the uninfected control (group A) before Day 13 (Figure 8C). IL-10 is a product of inflammatory responses and it is a component of an activity-dependent negative-feedback loop, which plays a significant role in inflammation control [8]. Expression of IL-10 in the re-epithelial wound tissue has a close correlation with the inflammation degree. Photomicrographs in Figure 8B presented the situations of IL-10 expression in the tissues of day 9. Apparently, the *K. p*-infected group (group B) expressed deepest yellow stains, indicating a large quantity of activated IL-10. In contrast, the uninfected group and the infected groups treated with antibiotics and B-2Ta displayed lighter stain. The trend was displayed in statistical analysis (Figure 8 D). The *K. p*-infected control group (group B) indicated a highest expression of IL-10 during the whole process, which was approximately twice higher than other groups at Day 9 and Day 13. The 10×MIC B-2Ta-treated group (group D) expressed less IL-10 on Day 9 than 2×MIC gentamicin-treated group (group C), and displayed an equal level expression of IL-10 and on Day 13, which represented that the 10×MIC B-2Ta exhibited an even better performance in neutralising the inflammation of *K. p* than 2×MIC gentamicin.

## DISCUSSION

Wound healing is a synergistic process, which involves various cells, cytokines and growth factors of the internal environment, while external factors can also affect the healing process. There are three indicators of inflammatory response: inflammatory inducers (infection or tissue damage), inflammatory sensors (mast cells and macrophages), and inflammatory mediators (cytokines and chemokines) [9]. The open dermal wounds are susceptible to the aerobic bacteria which can cause infections in the outer layers of skin, the infections could extend into subcutaneous layer, and subsequently alter the wound microenvironment and impair epithelialisation of the wound. Some clinical bacteria usually acquire additional mutations to resist the effects of conventional antimicrobial agents. Additionally, the microorganisms could also survive in the biofilm in wounds, in this case, practical therapeutic approaches are required, even if the mechanisms of infection are controversial [10]. In this *in vivo* study, we employed a model which debrides the biofilm and retains the infected granular tissue on the rats, and we treated the wound by using a novel identified B-2Ta peptide to assess the efficiencies of this antimicrobial agent.

B-2Ta is a short antimicrobial peptide which contains a cysteine disulphide bond whose specific amphipathic alpha-helix structure is currently perceived as being involved in interaction with the well-defined lipid bilayers of bacterial outer membranes. The current proposed theories about how AMPs lead to microorganism damage are ‘barrel-stave’, ‘carpet’ and ‘toroidal-pore’ modes, which may facilitate the formation of pores and lysis of the bacterial cellular outer membranes. In this study, we performed 10×MIC B-2Ta concentration administration, which aimed to minimise the differences of the efficacy between animals, and enhance the killing efficiency of the peptide [11, 12]. The SEM photographs showed the regular shaped holes on the *K. p* cell membranes when treated with a high-dosage of B-2Ta, confirming the structural deficiency on bacteria caused by AMP *in vivo*. The bacteria and bacteria-free serum are collected from the animal rather than lab-conserved materials to maintain the authenticity and consistency of the experimental design. In consideration of many impact factors, which can influence the treatment efficiency, therefore 10×MIC concentration was used on the wounded rats. Of note, if the peptide B-2Ta is going to be developed for clinical application, the haemolysis needs to be further decreased. Amino acid substitutions in the primary structure of the peptide is a commonly approach to balance the net charged and hydrophobicity in B-2Ta, which is essential to achieve the best antimicrobial activity while minimising red blood cell lysis, and warrant further investigations [13].

In this study, Figure 4 showed a remarkable acceleration of wound closure rate in the high-dosage of the B-2Ta-treated group in the period of day 9 to day 13. The apparent outcomes indicate the improvement efficiency of B-2Ta in the wound healing process. The intrinsic mechanisms might be directly promoting the wound healing process. Inflammation is a cellular protective response to pathogens, infection or tissue damage. In the early stage of wound healing, the primary immature granulation tissue is forming with inflammatory cells, angioblasts, fibroblasts, collagen fibres and new blood vessels, which are essential in the later stage in repairing the wound and composing mature granulation tissues [14]. The neovascularisation process launches from the proliferative phase as early as the third day of creating the injuries, involving endothelial cell proliferation and vessel formation [15]. The infected wound environment commonly has reduced release of angiogenic growth factors and inhibited angiogenesis process. We probed the CD 31 (pecam-1) on day 4, day 9 and day 13 as the indicator to assess the wound angiogenesis capability, and we quantified vessel distribution through the tissue sections. CD31 is a common signalling molecule expressed among the vascular compartment, which is known to have diverse roles in vascular biology including angiogenesis, platelet function, and thrombosis [16]. Previous reports found that the expression of CD31 is reduced in endothelial cells upon the stimulation of tumour necrosis factor (TNF)-alpha [17], and under inflammatory conditions, CD31 is downregulated. CD31 is also viewed as a protective molecule under certain acute and chronic inflammatory conditions, such as in collagen-induced arthritis [18, 19] and pulmonary hyperoxia [20]. In uninfected rats, the wounds expressed higher levels of CD 31 than all infected rats on day 4 and day 9, until the CD 31 expression levels in 10×MIC B-2Ta-treated rats became higher than other groups on day 13. This phenomenon suggested that the angiogenesis process was protected by the treatment of B-2Ta since the infection has been controlled in the late healing period. Additionally, we assessed the inflammatory levels of wounds by employing interleukin (IL)-10 as an indicator. IL-10 is an anti-inflammatory cytokine produced by various of myeloid and lymphoid cells, and the main sources are regulatory T cells, dendritic cells and macrophages depending on the ligations or stimulation between the metrocyte and upstream factors. The upregulation of IL-10 is stimulated by TLR4, TRAF3, NF-κBp65/p50 and ERK kinase modulated cell signalling. As an important anti-inflammatory cytokine [21], IL-10 inhibits the activity of Th1, Th2 and CD8+ T cells during the infection procedures. It restrains the pathogen clearance and mediates the immunopathology [22]. During bacterial infection, IL-10 down-regulates Th1 by suppressing overexpressed immunopathology associated factors, such as IFN-γ and TNF-α, and subsequently preventing multiple severe immune responses [8, 23]. It also synergistically regulates IL-4, IL-5, and IL-13 to alleviate severe fibrosis [24]. The uninfected rats expressed minimal IL-10 among the four groups, while the *K. p*-infected control released large amounts of IL-10 during the whole experiment period, reflecting a serious condition of inflammatory substances and factors in the wounds. Although we hypothesise that the peptide B-2Ta inhibited or kill the bacterial though a membrane disruption mechanism, we don’t rule out the possibility that B-2Ta could partially exert its function via activating IL-10 signalling to trigger immune response locally. In another aspect, time scale measurements between the three infected groups indicated that the bacterial infection was not improved in the early and middle healing stages.

In this study, it was verified that B-2Ta, as an AMP, effectively restrained *K. p* growth through damaging the integrity of microorganism structures in the wound environment, improved the bacterial infection status and accelerated the angiogenesis and granulation tissue maturing process. On the cellular level, reduction of IL-10 demonstrated that B-2Ta could relieve inflammation by targeting the bacterial infection. In conclusion, B-2Ta promoted angiogenesis and re-epithelialisation in *K. p*-infected rats in a comparable rate with conventional antibiotics, which provided evidence and clues for further pre-clinical studies of B-2Ta as a novel antimicrobial agent.

## MATERIALS AND METHODS

### Specimen biodata, animals and *Klebsiella pneumoniae* strain

*Pelophylax* kl. *esculentus* (n=30) were obtained from a commercial source. The frogs were adults and settled for three months before secretion harvesting. Skin secretions were obtained from the dorsal skin by mild transdermal electrical stimulation (4 ms pulse width, 50 Hz, 5 V) for 30s [25] prior to collected by rinsing with distilled deionized water, and then subjected to snap frozen with liquid nitrogen, lyophilised and stored at −20°C for future work.

Three-month aged male Sprague-Dawley (SD) rats were obtained from the Experimental Animal Centre of Tianjin Medical University (China). Rats were housed in individual plastic cages lined with wood shavings, maintained on a 12:12 h dark-light cycle, and fed standard laboratory rat chow (2018 Teklad Global 18% Protein Rodent Diet; 3.1 kcal/g, Shardlow Teklad, UK) and tap water ad libitum, unless otherwise specified. All animal experiments were approved by the Animal Care Committee of the National Natural Science Foundation of China, project 81403394. The present protocol was approved by the host institutional animal care committee.

The *Klebsiella pneumoniae* (*K. pneumoniae* or *K. p*) bacteria sample was collected from the wound secretion of a diabetic gangrenous (DF) patient in the First Affiliated Hospital of Tianjin university of Traditional Chinese Medicine. The secretion was cultured and adjusted primarily, and the target bacteria group was analysed by a Vitek 60 bacterial identification systems (Bio-Merieux, France). The *K. p* group was diluted into Phosphate Buffered Saline (PBS) and measured under 550 nm until 10^8^ CFU/ml were obtained. During the *in vivo* experiments, the rat wound secretions sampled at specific time points were cultured in LB medium at 37°C for 18h, for assessing the microbial sensitivity.

### Molecular cloning and primary structural analysis of novel peptide in the skin secretion of *pelophylax* kl. *esculentus*

The novel peptide B-2Ta was isolated and identified from the skin secretion of *Pelophylax* kl. *esculentus* by using “shotgun” cloning with a degenerate sense primer (S1; 5′-GAWYYAYYHRAGCCYAAADATGTTCA-3′) (R=A/G; Y=C/T; H=A/T/C; W=A/T; D=G/A/T) and a NUP primer (supplied with the kit) as described by the manufacturer (Clontech UK) [26]. LCQ-Fleet™ electrospray ion-trap mass spectrometer was used to confirm the authenticity of the primary structure of the novel peptide from the frog skin secretions [26, 27].

### Peptide synthesis and secondary structure prediction analyses

Once the primary structure was unequivocally confirmed through both mass spectrometry fragmentation sequencing and molecular cloning strategy, the solid-phase Fmoc chemically approach was adopted via Tribute™ automated solid-phase peptide synthesiser 4 (Protein Technologies, Tucson, AZ, USA) to synthesise the novel peptide. After that, the secondary structure prediction was performed by using SWISS-MODEL workspace and HeliQuest for alpha-helix modelling [3, 28–31]. The PyMOL visualisation system established a 3D mimic pattern of the short peptide.

### Antimicrobial assay

The antimicrobial activity was evaluated by MIC assay and MBC assay by using four microorganisms: The Gram-positive bacterium, *Staphylococcus aureus* (*S. aureus*) (NCTC 10788), the Gram-negative bacterium, *Escherichia coli* (*E. coli*) (NCTC 10418) and the *Klebsiella pneumoniae* (*K. p*) (collected from the First Affiliated Hospital of Tianjin university of Traditional Chinese Medicine in project 81403394 of National Natural Science Foundation of China), and the yeast, *Candida albicans* (*C. albicans*) (NCPF 1467). These four microorganisms were grown in Mueller-Hinton Broth (MHB) (Oxoid, Basingstoke, Hampshire, UK) at 37°C until logarithmic phase growth. The optical densities (ODs) of culture media were detected at 550 nm to assess dilution factors required to achieve inoculation concentrations of 1×10^6^ CFU/mL for bacteria or 5 ×10^5^ CFU/mL for the yeast. Tested peptide concentrations were prepared from 512 to 1 mg/L before loaded and mixed in a 96-well microtiter plate with culture medium for 18h incubation at 37°C. The MICs were determined as the lowest concentration of peptide where no growth was detectable using an ELISA plate reader (Biolise BioTek EL808, Winooski, VT, USA). After which, 10 μL of medium from each well was inoculated onto Mueller Hinton Agar (MHA) (Oxoid, Basingstoke, Hampshire, UK) plates to test the MBC. The MBC value is defined as the lowest peptide concentrations from which no colonies were grown on the MHA plates.

### Haemolysis assay

The haemolysis assay was performed using erythrocytes prepared from defibrinated horse blood (TCS Biosciences Ltd., Buckingham, UK). Two hundred μL of 4% (v/v) suspension of erythrocytes in PBS were incubated with peptide gradient prepared in PBS from concentration of 512 to 1 mg/L at 37°C for 2 h. Lysis of erythrocytes was assessed by measurement of optical density at 550 nm using the ELISA plate reader. Negative controls and positive controls were PBS alone and PBS containing Triton X-100 (Sigma-Aldrich, St. Louis, MO, USA), respectively.

### Wound induction and *Klebsiella pneumonia* infection

Rats were anesthetised with intraperitoneal injection of 3% Nembutal (1 ml/kg), and the dorsal areas were depilated using a shaver and soap-suds. A 2-cm wound was created on the back of each rat with a dermal biopsy punch. The wounds were treated distinctively depending on the groups and covered with Tegaderm (3M, Minneapolis, MN) to keep them moist and maintain consistency. 32 male SD rats were equally divided into four groups which were A) uninfected control, B) *K. p*-infected control, C) *K. p*-infected group with 2×MIC gentamicin-treatment, D) *K. p*-infected group with 10× MIC B-2Ta treatment. The infection of wounds was achieved by inoculation with 20 ul of bacterial suspension (CFU 10^8^) and kept one day without treatment for proliferation. Alginate powder was dispersed in distilled water under vigorous stirring (1500 rpm) for 15 min then the solution was left under gentle stirring (100 rpm) for 2 h. Finally polymer solutions were left motionless for 4 h to remove air bubbles [32], B-2Ta peptide loaded gels were prepared by mixing in an orbital shaker a precise volume of alginate solution to obtain a final peptide concentration of 10 x MIC in alginate solution at 5.0% (w/w). Daily parallel treatments were cleaning purulent exudate, cover the gel onto the wound and changing the bio-dressing. The grouping and treatment procedures are described in Table 2. During the 14-day period of treatments, the model rats were separately fed in isolation to avoid cross infection. To observe the wound healing conditions at a molecular level and in morphology, each of 8 animals in the same group were treated with two schemes: the first 5 rats were sampled using a quarter of tissues from different wound areas on days 4, 7, 9 and 13, which served for trials of morphology, immunohistochemistry and proteomic studies; the remaining 3 rats were treated with the same method and their dorsal areas were photographed instead of cutting the tissues, which was for wound healing assessment. After the final treatment on day 14, all the animals were euthanised using ethyl carbamate. The treatment plan is described in Supplementary Table 1. All assessments were blinded to treatment.

**Table 2:** Treatment of groups A, B, C and D according to the *in vitro* MIC assay

Groups	*Conditions*	No.	Wound	Infection	Reagents	Treatment
**A**	Untreated control	1-8	√	×	Normal saline 500 mL	Clean purulent exudate
**B**	Infection control	9-16	√	√	Normal saline 500 mL	Add reagent
**C**	2×MIC gentamicin	17-24	√	√	Gentamicin (2×MIC 500 mL)	Change the dressing
**D**	10×MIC B-2Ta	25-32	√	√	B-2Ta (10×MIC 500 mL)	

**Note:** The MIC values of gentamicin and B-2Ta were 32 mg/L and 64 mg/L against *K. p*, the inoculation concentration of group C and group D were 64 mg/L and 0.64 mg/ml, respectively.

### Bacterial sampling in wound area for growth assessment

During the 14-day treatment, the microbes derived from the wound area were cultivated for assessing bacterial inhibition abilities. Briefly, exudate samples were acquired by fully swabbing moist wounds and diluted into 10 ml and 100 ml PBS as suspensions. The bacteria were daubed onto LB solid media and cultivated overnight to check if some colonies will be cultivated on the plate. To count live *K. p* in wound areas, a cylindrical punch with a stopping piston was used. Skin samples were taken at three sites in the wound at day 1, 4, 9, 13 post infection. The biopsies were taken at its deep base and they were weighted, homogenised and diluted. The quantity of bacteria was determined as CFU / grams (CFU/g).

### Sample preparation and scanning electron microscopy (SEM)

The morphological changes of bacteria after B-2Ta treatment was assessed by SEM which followed the protocol published by Chen [33]. Briefly, one ml of *K. p* suspension (10^8^ CFU/mL) was used to mix with 1 ml B-2Ta peptide solution to reach a final concentration of 128 mg/L and cultivated overnight at 37°C. The untreated control was prepared in salt-free Lysogeny broth (LB) medium only. After the cultivation, the suspensions were condensed by centrifugation at 4000 x g for 10 min at 4°C and then fixed with 2.5% glutaraldehyde at 4°C for 2 h. After that, the cells were dehydrated by sequential treatment with 30, 50, 60, 70, 80, 90 and 100% ethanol for 10 min each time. Then, a drop of 8 μl dehydrated bacterial cell suspension was transfer to a silica plate to air dried before mounted. The mounted samples were sputter-coated with gold (10s, 50 mA) for observation under a SEM (SU8010, Hitachi) at an accelerating voltage of 10 kV.

### Wound closure, re-epithelialisation and angiogenesis assessment

The dorsal wounds of rats were recorded by images when they were created (day 0) and before treating at each time. The wound area was measured using photo shot software (Scion, Frederick, MD), and the percentage of the re-built wound areas was calculated by normalised with initial wounding area on day 0 by using GraphPad Prism 6.0.1 (San Diego, California, USA). The re-epithelialisation condition was assessed by a morphological and histological study using tissue sections stained with haematoxylin and eosin (H&E) [34]. For angiogenesis assessment, the tissue slices were immunostained for platelet/endothelial cell adhesion molecule-1 (Pecam-1), also known as cluster of differentiation (CD) 31 [35].

### Immunohistochemical analyses

Paraffin-embedded sections were de-paraffinized and placed in water. The endogenous peroxidase activity was blocked using 3% H_2_O_2_ in methanol [36]. With brief rinsing in PBS (PH7.4) (5 min×2), the sections were subjected to antigen retrieval in citric acid buffer and microwave heating for 3 min × 2, and serum blocking in 5% bovine serum albumin (BSA) (Boster, Wuhan, China, Ref. AR0004) for 10 min, and incubated in 200 μg/ml of rabbit polyclonal anti-rat IL-10/CD31 (Boster, Wuhan, China, Ref. BA1645-1/BA1201-1/BA2966) in 0.1% BSA-PBS overnight at 4°C and rinsed in PBS. The secondary antibody and Biotin-SP (Boster, Wuhan, China, Ref. SA1055) were applied following the manufacturer’s instructions. Sections were rinsed in PBS, stained with 3-Amino-9-ethylcarbazole (AEC), rinsed again and counterstained with haematoxylin. The histomorphology and gray value of the protein expression were analysed through Image-Pro Plus 6.0 software (Media Cybernetics, Inc.).

### Statistical analysis

Statistical analysis was performed by using ANOVA (Bonferroni test) with the SPSS statistical software 18.0 (SPSS Inc., Chicago, IL, USA). All results are presented as mean ± SD, the error bars represent the standard error of mean (SEM). Significances were defined when P value<0.05.

## SUPPLEMENTARY MATERIALS FIGURES AND TABLES



## Abbreviations

B-2Ta: Brevinin-2Ta
AMP: antimicrobial peptide
MIC: minimal inhibitory concentration
MBC: minimal bactericidal concentration
TFA: trifluoroacetic acid
SD rat: Sprague-Dawley rat
PBS: phosphate buffered saline
*K. p* or *K. pneumoniae*: *Klebsiella pneumonia*
RACE: rapid amplification of cDNA ends
PCR: polymer chain reaction
NUP: nested universal primer
HPLC: High-performance liquid chromatography
DMF: dimethylformamide
HBTU: 2-(1H-benzotriazol-1-yl)-1,1,3,3-tetramethyluronium hexafluorophosphate
TIPS: Triisopropylsilane
MHB: Mueller-Hinton Broth
CFU: colony forming units
MHA: Mueller Hinton Agar
LB medium: Lysogeny broth medium
OD: optical density
SEM: scanning electron microscopy
H&E: haematoxylin and eosin
IHC: Immunohistochemical
MS: mass spectrometry
IL: interleukin
MALDI-TOF-MS: matrix-assisted laser desorption/ionisation time-of-flight mass spectrometry
BSA: bovine serum albumin
