# Supplementary material for: Assessment of antimicrobial and wound healing effects of Brevinin-2Ta against the bacterium Klebsiella pneumoniae in dermally-wounded rats
Source: Oncotarget. 2017 Nov 30;8(67):111369–85. doi: 10.18632/oncotarget.22797 (PMC5762328; doi:10.18632/oncotarget.22797)
Supplement: Supplementary file 1 [file oncotarget-08-111369-s001.pdf]

Assessment of antimicrobial and wound healing effects of Brevinin-2Ta against the bacterium *Klebsiella pneumoniae* in dermally-wounded rats

SUPPLEMENTARY MATERIALS

```
       M  F  T  L   K  K  S   L  L  L   F  F  F  L   G  T  I
   1ATGTTCACCT TGAAGAAATC CCTGTTACTC TTTTTCTTTC TTGGGACCAT
    TACAAGTGGA ACTTCTTTAG GGACAATGAG AAAAAGAAAG AACCCTGGTA
     S  L  S   L  C  Q  E   E  R  N   A  D  E   D  D  G  E
  51CTCCTTATCT CTCTGTCAGG AAGAGAGAAA TGCTGATGAG GACGATGGGG
    GAGGAATAGA GAGACAGTCC TTCTCTCTTT ACGACTACTC CTGCTACCCC
      M  T  E   E  E  K   R  G  I   L  D  T  L   K  N  L
 101AAATGACAGA GGAAGAAAAA AGAGGTATCC TGGATACGCT GAAGAATTTA
    TTTACTGTCT CCTTCTTTTT TCTCCATAGG ACCTATGCGA CTTCTTAAAT
     A  K  T  A   G  K  G   I  L  K   S  L  V  N   T  A  S
 151GCCAAGACAG CAGGCAAAGG TATACTGAAG AGTCTGGTGA ATACGGCATC
    CGGTTCTGTC GTCCGTTTCC ATATGACTTC TCAGACCACT TATGCCGTAG
     C  K  L   S  G  Q  C   *
 201TTGTAAACTT TCTGGACAAT GCTAAAACAT GAATTGGAAG TCATTTGATG
    AACATTTGAA AGACCTGTTA CGATTTTGTA CTTAACCTTC AGTAAACTAC
 251CAGCATATCA TTTAGCTAAA TACTAAATGT CTGATAAAAA ATAAAAATAT
    GTCGTATAGT AAATCGATTT ATGATTTACA GACTATTTTT TATTTTTATA
 301CACATGAAAA AAAAAAAAAA AAAAAAAAAA
    GTGTACTTTT TTTTTTTTTT TTTTTTTTTT
```

Supplementary Figure 1: Nucleotide and translated amino acid sequences of cDNA cloned from skin secretion library of *Pelophylax* kl. *esculentus* which encoded B-2Ta. The putative signal peptide is double-underlined, the mature peptide is single-underlined and the stop codon is indicated by an asterisk.

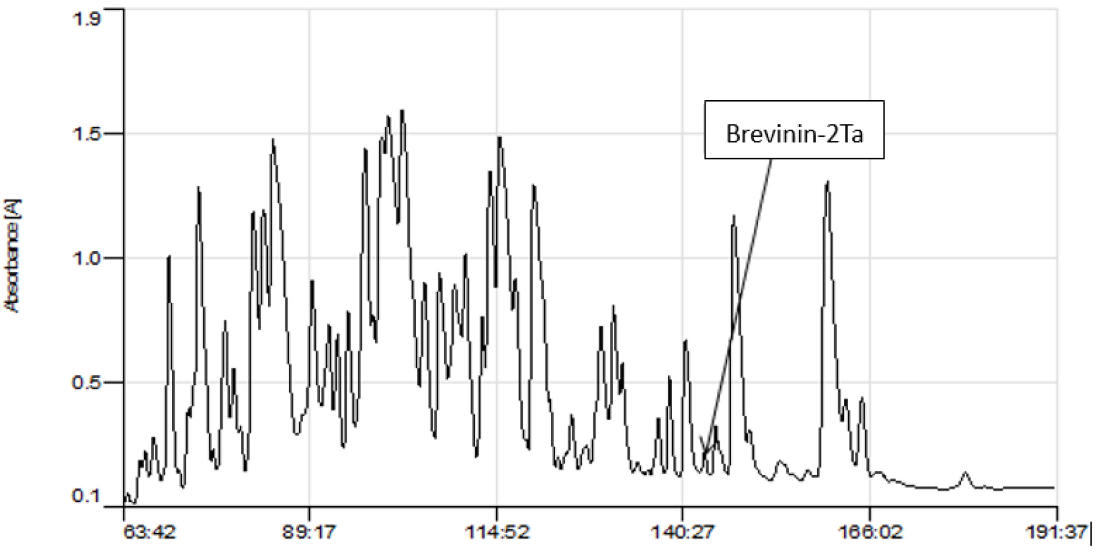

Supplementary Figure 2: Regions of rp-HPLC chromatogram of *Pelophylax* kl. *esculentus* skin secretion. The Y-axis represents the relative absorbance at wavelength 214 nm and the X-axis represents the retention time in minutes. The arrow indicates the elution position/retention time of B-2Ta.

#1	b(1+)	b(2+)	b(3+)	Seq.	y(1+)	y(2+)	y(3+)	#2
1	58.02875	29.51801	20.01443	G				33
2	171.11282	86.06005	57.70912	I	3296.78188	**1648.89458**	**1099.59881**	32
3	284.19689	142.60208	95.40381	L	3183.69781	1592.35254	**1061.90412**	31
4	399.22384	200.11556	133.74613	D	3070.61374	**1535.81051**	1024.20943	30
5	**500.27152**	250.63940	167.42869	T	2955.58679	**1478.29703**	**985.86711**	29
6	**613.35559**	307.18143	205.12338	L	2854.53911	**1427.77319**	**952.18455**	28
7	741.45056	371.22892	247.82170	K	2741.45504	**1371.23116**	**914.48986**	27
8	**855.49349**	428.25038	285.83601	N	2613.36007	**1307.18367**	871.79154	26
9	968.57756	484.79242	323.53070	L	2499.31714	**1250.16221**	**833.77723**	25
10	**1039.61468**	**520.31098**	347.20974	A	2386.23307	**1193.62017**	796.08254	24
11	**1167.70965**	**584.35846**	389.90807	K	2315.19595	**1158.10161**	772.40350	23
12	**1268.75733**	**634.88230**	423.59063	T	2187.10098	**1094.05413**	729.70518	22
13	**1339.79445**	**670.40086**	447.26967	A	2086.05330	1043.53029	696.02262	21
14	**1396.81592**	**698.91160**	466.27682	G	2015.01618	**1008.01173**	**672.34358**	20
15	$1524.9^{108}9$	**762.95908**	508.97515	K	1957.99471	**979.50099**	653.33642	19
16	1581.93236	**791.46982**	527.98230	G	**1829.89974**	**915.45351**	610.63810	18
17	**1652.96948**	**826.98838**	551.66134	A	1772.87827	886.94277	591.63094	17
18	1766.05355	883.53041	589.35603	L	**1701.84115**	851.42421	**567.95190**	16
19	1894.11213	947.55970	**632.04223**	Q	**1588.75708**	**794.88218**	530.25721	15
20	1981.14416	**991.07572**	661.05290	S	**1460.69850**	730.85289	487.57102	14
21	2094.22823	**1047.61775**	**698.74759**	L	1373.66647	687.33687	458.56034	13
22	2207.31230	**1104.15979**	**736.44228**	L	1260.58240	630.79484	420.86565	12
23	2321.35523	**1161.18125**	774.45659	N	**1147.49833**	574.25280	383.17096	11
24	2458.41414	**1229.71071**	**820.14290**	H	**1033.45540**	517.23134	345.15665	10
25	2529.45126	**1265.22927**	843.82194	A	**896.39649**	448.70188	299.47035	9
26	2616.48329	**1308.74528**	**872.83261**	S	**825.35937**	413.18332	275.79131	8
27	2719.49248	**1360.24988**	**907.16901**	C	**738.32734**	369.66731	246.78063	7
28	2847.58745	1424.29736	949.86733	K	**635.31815**	318.16271	212.44423	6
29	2960.67152	**1480.83940**	**987.56202**	L	507.22318	254.11523	169.74591	5
30	3047.70355	1524.35541	**1016.57270**	S	394.13911	197.57319	132.05122	4
31	3104.72502	**1552.86615**	**1035.57986**	G	307.10708	154.05718	103.04054	3
32	3232.78360	1616.89544	**1078.26605**	Q	250.08561	125.54644	84.03339	2
33				C	122.02703	61.51715	41.34719	1

Supplementary Figure 3: Predicted singly-charged, doubly-charged and triply-charged b-and y-ions following MS/MS fragmentation of B-2Ta. Observed ions are highlighted in bold and underlined.

Supplementary Table 1: The treatment and sample preparation in the period of 14 days for antimicrobial, morphological, and immunohistochemical analyses

		**Day 1**<br>1. Dressing removed<br>2. Photography<br>3. Microbial cultivating<br>4. Cutting up tissue sample<br>5. Tegaderm® dressing		**Day 9**<br>1. Dressing removed<br>2. Photography<br>3. Microbial cultivating<br>4. Cutting up tissue sample<br>5. Tegaderm® dressing	
Time point (Day)	0	1	4	9	13
	**Day 0**<br>1. Rats weighted<br>2. Rats wounded<br>3. Photography<br>4. Treatment applied (500μl)<br>5. Tegaderm® dressing		**Day 4**<br>1. Dressing removed<br>2. Photography<br>3. Microbial cultiating<br>4. Cutting up tissue sample<br>5. Tegaderm® dressing		**Day 13**<br>1. Dressing removed<br>2. Photography<br>3. Microbial cultivating<br>4. Cutting up tissue sample<br>5. Rats execution

Note: The four groups were conducted of uniform treating strategies.

Supplementary Table 2: Score of morphological features

(A) Standard score

Score	Re-epithelialization	Granulation tissue	Collagen deposition
0	Hardly to see	Hardly to see	Hardly to see
1	Trace and focal migrating	Trace	None
2	Trace and focal migrating	Hypocellular and no vessels	Trace
3	Partial	Many cells and few vessels	Slight
4	Hypertrophic and partial stratum corneum	Many fibroblasts, some fibers and some vessels	Moderate

(B) Wound-healing score after different recover time

Groups	1 day after damage	4 days after damage	9 days after damage	13 days after damage
Uninfected Control	0.5	1.5	3	4
Infected Control	0	1	2	2
2×MIC gentamicin treatment	0	1.5	3.5	4
10×MIC B-2Ta treatment	0.5	1	3	3.5
